# Public School Teachers’ Nutrition Knowledge and Perceptions of the School Food Environment in Kazakhstan

**DOI:** 10.3390/nu18071042

**Published:** 2026-03-25

**Authors:** Svetlana Rogova, Olzhas Zhamantayev, Olga Plotnikova, Denis Turchaninov, Zhanna Yesmagambetova, Nurbek Yerdessov, Marat Kalishev

**Affiliations:** 1 School of Public Health, Karaganda Medical University, Karaganda 100000, Kazakhstan; s.rogova@qmu.kz (S.R.);; 2 Department of Occupational Hygiene and Occupational Pathology, Omsk State Medical University, Omsk 644099, Russia; 3 Karaganda Regional Branch of the “Salidat Kairbekova National Scientific Center for Health Development” of the Ministry of Health of the Republic of Kazakhstan, Karaganda 100000, Kazakhstan

**Keywords:** school food environment, teachers, nutrition knowledge, health education, school children, public health policy, Kazakhstan

## Abstract

**Background/Objectives**: Schools, as structured social environments, are important settings for shaping lifelong eating habits, and teachers play a mediating role in nutrition education. This study aimed to assess nutrition knowledge among public school teachers, examine their perceptions of the school food environment, and identify factors associated with knowledge scores. **Methods**: A stratified cross-sectional survey was conducted among 736 teachers from 12 public schools during the fall of 2025. A structured questionnaire based on the Knowledge–Attitudes–Practice model was used to evaluate nutrition knowledge, teaching practices, perceived school food environment, and teachers’ observations of student food-related behaviors. Group differences were examined using *t*-tests and ANOVA, and multivariable logistic regression was applied to identify factors associated with low nutrition knowledge. **Results**: The mean knowledge score was 6.26 ± 2.64 out of 12, with 23.6% of teachers classified as having low knowledge, 59.9% satisfactory, and 16.4% good. Primary school teachers scored significantly higher than subject teachers (7.27 vs. 5.64; *p* < 0.001). Regular conduct of nutrition classes was associated with lower odds of low knowledge (adjusted OR 0.10, 95% CI 0.05–0.23, *p* < 0.001). A sensitivity analysis using continuous knowledge scores confirmed this pattern, with the absence of nutrition teaching predicting a 1.40-point reduction in scores (95% CI −1.86 to −0.94, *p* < 0.001). Teachers rated school meal quality moderately high, and frequently observed student refusal of canteen food and purchase of sweets or fast food outside school. **Conclusions**: This study found that public school teachers in Karaganda, Kazakhstan showed satisfactory overall nutrition knowledge, with gaps in applied understanding and common dietary myths. Primary teachers and those actively teaching nutrition had higher knowledge scores, indicating an association between pedagogical engagement and content knowledge. To help optimize the school food environment, educational policies would benefit from the development of cross-curricular instructional materials fitted specifically for secondary school subject teachers.

## 1. Introduction

Healthy and balanced nutrition during childhood and adolescence forms the foundation for normal physical, cognitive, and emotional development [1,2,3,4]. However, over the past few decades, the world has witnessed a steady rise in the prevalence of diet-related diseases. Overweight and obesity among children and adolescents are increasingly becoming one of the most pressing public health issues, driving up healthcare costs, reducing quality of life, and elevating the risk of chronic conditions in adulthood [5,6,7]. Kazakhstan is no exception to this trend. According to projections from the World Obesity Federation, the number of children with obesity in the country could exceed half a million by 2030 [8,9].

School nutrition in Kazakhstan is regulated through national sanitary and nutritional standards for educational institutions, which are aligned with recommendations of the World Health Organization [10]. These standards define recommended food groups, portion sizes, and limits for sugar, salt, and saturated fat in children’s meals and function as the practical application of national Food-Based Dietary Guidelines in school settings. They directly shape the composition of school meals and the institutional expectations for nutrition education, providing an important contextual framework for interpreting teachers’ nutrition knowledge and perceptions.

In this context, schools play an important and prominent role. They are not merely places of learning but social environments where habits and behavioral patterns, including attitudes toward food, are shaped daily [11,12]. Teachers emerge as key figures, because their personal example, involvement in school initiatives, and willingness to discuss health matters directly influence how children perceive nutrition. The success of educational and preventive programs largely hinges on educators’ professional training, motivation, and engagement [13,14]. In Kazakhstan, the fundamentals of healthy nutrition are embedded within the expected learning outcomes of the State Compulsory Educational Standard for Primary Education [15]. These concepts are primarily implemented through cross-curricular integration and various extracurricular educational formats; consequently, the consistency and depth of instruction can vary significantly across schools and classrooms. To support these educational objectives, various informational and pedagogical resources are available for both students and parents, including digital tools such as the Balaman.kz platform [16]. In practice, however, the successful delivery of this content relies heavily on the individual educator’s professional readiness and the extent to which they systematically incorporate nutrition topics into both curricular and extracurricular activities. At the same time, studies indicate that many teachers’ knowledge of nutrition principles remains limited, with practical skills often fragmented [17,18]. Modern schools operate in an information landscape where reliable scientific data on nutrition coexist with myths and conflicting claims [18,19]. Although family environments undoubtedly exert the strongest influence on long-term eating habits, schools provide complementary structured opportunities where teachers can reinforce healthy behaviors through daily interaction and educational initiatives [20].

In Kazakhstan, and especially in major industrial centers such as Karaganda, balanced nutrition among children and adolescents remains an important public health issue. The urban environment in these cities shows marked heterogeneity in infrastructure development, including access to public catering facilities and the density of fast-food outlets [21,22]. This heterogeneity creates differing contexts for schools across administrative districts when implementing nutrition-related initiatives, which calls for examination of teachers’ perceptions of school meals and related pedagogical practices at the district level. Under these conditions, local studies are especially informative, as they allow characterization of the school food environment and assessment of teachers’ readiness to engage in nutrition education, while also identifying school-level barriers and available resources across city districts.

Despite growing interest in cultivating a culture of healthy eating in schools, empirical evidence from Central Asia remains limited, particularly regarding the professional readiness of teachers to support nutrition initiatives. Most available studies focus either on student dietary patterns or on regulatory aspects of school feeding, while less attention has been given to the human component of school food systems. Teachers function not only as transmitters of curriculum content but also as daily role models, supervisors of meal times, and mediators between school policy and family practices. Their knowledge and perceptions may influence how consistently nutrition standards are implemented in everyday school life. Within this context, the Knowledge–Attitudes–Practice (KAP) framework provides a structured lens for examining teachers’ roles [23,24]. Knowledge reflects cognitive preparedness, attitudes capture perceived institutional influence and responsibility, and practices represent enacted educational behaviors. Rather than treating these domains as isolated constructs, this study conceptualizes them as interacting components within the school nutrition environment. In particular, we examine whether engagement in nutrition-related teaching practices is associated with higher knowledge levels, and whether contextual factors such as district and professional experience shape these patterns.

This study aimed to assess nutrition knowledge among public school teachers in Kazakhstan, evaluate their perceptions of the school food environment, and identify factors associated with variations in their nutrition knowledge. We hypothesized that active pedagogical engagement with health curricula would be positively associated with higher nutrition knowledge scores. By situating teacher competencies within a broader school food system perspective, this study contributes empirical data from a region with limited published evidence in international nutrition education research.

## 2. Materials and Methods

### 2.1. Study Design and Participant Selection

#### 2.1.1. Study Protocol

This stratified cross-sectional study was conducted among educators in public general education schools in Karaganda, Kazakhstan. Data collection took place from September through November 2025. The study was organized through direct site visits to the participating educational institutions. During these visits, the research team held briefing meetings with school administrations to outline the study objectives, procedural requirements, and ethical safeguards. School leadership subsequently informed the teaching staff of the opportunity to participate. All schools included in the study provide organized meals through on-site cafeterias as required under national sanitary and nutritional standards for educational institutions. School meal provision is universal for primary school students and available to secondary students across all participating schools, ensuring that all teachers had the opportunity to observe school feeding practices irrespective of their teaching level or district.

To ensure voluntary involvement and minimize disruption to the educational process, the questionnaire was administered electronically via an encrypted online platform Google Forms (Google LLC, Mountain View, CA, USA). Teachers completed the survey independently at their convenience. Before accessing the survey instrument, respondents were required to read a digital informed consent form. Confirmation of voluntary participation was mandatory to proceed. The study was conducted in accordance with the Declaration of Helsinki and approved by the Institutional Review Board of Karaganda Medical University (protocol code 2025-06-17/12, 17 June 2025). No personal identifiers were recorded, ensuring participant anonymity and data confidentiality.

#### 2.1.2. Inclusion/Exclusion Criteria

Eligibility criteria included full-time employment as a primary or subject teacher in one of the selected public schools with at least one year of teaching experience. Administrative staff, part-time educators, and those on leave during the data collection period were excluded. “Subject teachers” refer to secondary-level specialists teaching specific disciplines (e.g., mathematics, biology, history), who typically comprise approximately 70% of public school educators in Kazakhstan according to Ministry of Education staffing data [25]. They were included to allow comparison with primary school teachers, who deliver holistic education that more frequently incorporates health and nutrition topics.

#### 2.1.3. Sampling Strategy

Stratification by administrative district was employed to capture socioeconomic and infrastructural diversity across the city: we selected three major, socioeconomically diverse districts (Southeast, Novy Gorod, and Maikuduk). Four schools were purposively sampled from each district (12 in total), with attention to variations in school size, geographic location, and infrastructure. This approach balanced feasibility with generalizability, capturing districts that differed in urban infrastructure and food environment characteristics. The sampling strategy followed established guidelines for school-based health and education research, providing sufficient statistical power and adequate representation of teachers from both primary and secondary levels.

The average cluster size (number of teachers per school, m) was estimated at 60, based on official staffing data from the Karaganda City Department of Education for the 2024–2025 academic year. To account for the nested structure of the data (teachers within schools), we assumed a conservative intraclass correlation coefficient (ρ = 0.02), a value commonly reported in school-based health and behavioral studies when individual-level outcomes are moderately influenced by the school environment. The design effect (DEFF) was calculated as [26]:

DEFF = 1 + (m − 1)ρ = 1 + (60 − 1) × 0.02 = 2.18

The required number of school clusters was estimated using standard cluster sampling methodology, accounting for the design effect [26,27]:

K ≈ 1/((ME^2^ m)/(z^2^ p(1 − p) DEFF + 1/N)

where: DEFF = design effect; m = average cluster size (number of teachers per school); ρ = intraclass correlation coefficient; K = required number of school clusters; ME = margin of error; z = critical value corresponding to the desired confidence level; p = expected prevalence (proportion); N = total population size. Assuming an average cluster size of 60 teachers, an intraclass correlation coefficient of 0.02, a margin of error of ±5%, a confidence level of 95% (z = 1.96), and a conservative prevalence estimate of 0.50, the calculated design effect was 2.18. Applying these parameters yielded a required sample of approximately 12 schools. With an average of 60 teachers per school, this yielded a minimum sample size of n = K × m = 720 participants. Accounting for a potential 10–15% non-response rate (typical in population surveys), approximately 800 teachers were invited. Ultimately, 736 teachers completed the survey, representing a response rate of 92%.

### 2.2. Instrument, Theoretical Framework and Measure

The questionnaire was developed using the Knowledge–Attitudes–Practice framework as an organizing structure (please see Appendix A for the tool). It comprised four sections:

(1) professional characteristics (district/school, status as primary school teacher or subject teacher, teaching experience);

(2) attitudes and perceptions of school meals (subjective evaluation of cafeteria food quality on a 5-point scale, assessment of the school’s role in shaping dietary habits, perceptions of parental awareness, and preferences regarding improvement measures);

(3) nutrition-related pedagogical practices (delivery of nutrition lessons or class sessions, use of teaching materials, supervision or observation of student meals);

(4) knowledge of nutrition principles (10 items covering basic dietary recommendations, nutrient functions, diet-related diseases, and validity of common nutrition statements).

We defined the school’s role in shaping dietary habits as the perceived extent of institutional influence on students’ food choices, rated on an ordinal scale from very significant to no influence. Parental knowledge/awareness referred to teachers’ estimates of families’ knowledge of healthy eating principles and was scored as high, average, or low. Teachers were asked to rate perceived parental knowledge rather than report observed behaviors. This variable captures teachers’ subjective judgments and does not represent a direct assessment of parental nutrition knowledge. Within the KAP framework, this measure is interpreted as an attitudinal indicator that reflects teachers’ beliefs about the broader family context influencing students’ dietary behaviors.

Knowledge items were derived from national nutrition standards for educational institutions and aligned with World Health Organization food-based dietary guideline concepts [10,28,29]. The total knowledge score ranged from 0 to 12. Seven single-answer items were scored with 1 point each. One food group ranking item was awarded 1 point only when the respondent’s ranking exactly matched the reference order derived from national and WHO dietary guidelines. Two multiple-choice items with more than one correct answer were graded with 2 points each, with credit awarded only when all correct options were selected simultaneously and no partial credit applied. The complete answer key and scoring rules for each item are provided in Appendix A.

Content validity was supported through expert review by a practicing dietitian and a public health specialist, whose feedback informed refinement of item wording and alignment with national school nutrition standards. Face validity was assessed through pilot testing with a convenience sample of teachers not included in the main study, confirming item clarity and response option interpretability. Internal consistency of the full knowledge subscale was acceptable (Cronbach’s α = 0.70), consistent with exploratory instruments assessing heterogeneous nutrition knowledge domains [30]. Attitudinal and practice items were not designed to form unidimensional scales, and subscale-level reliability coefficients for these sections were therefore not calculated.

### 2.3. Statistical Analysis

Teachers’ knowledge of nutrition principles was scored from 0 to 12 based on correct responses to 10 items (Q11–Q20). For descriptive purposes, scores were categorized into three levels: low (0–4 points), satisfactory (5–8 points), and good (9–12 points). These thresholds were defined a priori by dividing the total score range into approximate tertiles, consistent with approaches used in comparable KAP-based studies [30], and were confirmed as interpretively reasonable during pilot testing. They represent descriptive categories for group-level analysis rather than externally validated clinical thresholds. For multivariable analysis, knowledge was dichotomized as low (0–4) versus satisfactory/good (≥5) to identify factors associated with the lowest knowledge tier, with the trade-off of reduced statistical power acknowledged.

To ensure the statistical robustness of the primary findings and address potential power loss associated with variable dichotomization, a sensitivity analysis was conducted. Specifically, we constructed a multiple linear regression model utilizing the continuous 12-point nutrition knowledge score as the dependent variable. Predictor variables entered into the model included pedagogical practice in nutrition, teaching position, years of work experience, and school location.

In addition to the continuous model, a secondary logistic regression utilizing an alternative cut-off threshold for the categorization of ‘low’ knowledge (score ≤ 5) was conducted to verify that the observed associations were not dependent on a specific dichotomization boundary.

We applied independent-samples *t*-tests for two-group comparisons (primary versus subject teachers) with Welch adjustment applied when Levene’s test indicated unequal variances and one-way ANOVA with Games–Howell post hoc tests for three-group comparisons (work experience categories, districts) to account for potential heteroscedasticity. Perceived parental knowledge ratings by school teachers were compared across groups using Pearson’s chi-square, and with Spearman’s rank-order correlation we assessed the relationship between perceived meal quality and nutrition knowledge score. Statistical significance was set at *p* < 0.05 for all tests and all analyses were two-tailed.

## 3. Results

A total of 736 teachers from 12 public schools in Karaganda, Kazakhstan, completed the survey (Table 1). Participants were drawn from three of the city’s largest districts (South-East, New City, and Maikuduk). Most respondents were subject teachers (456, 62.0%) and the rest were primary school teachers (280, 38.0%). Regarding experience, 34.4% (253) had ≤5 years, 36.4% (268) had 6–15 years, and 29.2% (215) had more than 16 years. Geographically, nearly half worked in New City (n = 355, 48.2%), followed by Maikuduk (n = 309, 42.0%) and South-East (n = 72, 9.8%).

### 3.1. Perceptions of School Nutrition Environment by Teachers

Teachers rated cafeteria meal quality on a 5-point scale (1 = very poor, 5 = excellent), with a mean score of 4.08 ± 0.57 (Table 2). The distribution was strongly positive-skewed, with 89.0% of ratings at 4 or 5. Primary school teachers rated quality significantly higher than subject teachers (4.26 ± 0.57 vs. 3.96 ± 0.54; Welch t(569.22) = 7.10, *p* < 0.001, Cohen’s d = 0.54).

Distribution of responses on student meal refusal frequency is shown in Table 2. Refusal patterns differed significantly by teacher position (χ^2^(4) = 31.06, *p* < 0.001): primary teachers more often reported “almost never” (21.1% vs. 9.6%) and less often selected “don’t know/don’t observe” (27.5% vs. 42.8%) compared to subject teachers. Differences were also significant by work experience (χ^2^(8) = 42.46, *p* < 0.001) and district (χ^2^(8) = 35.23, *p* < 0.001), with South-East district showing the highest rates of frequent refusal (22.2% “often” or “almost always”).

Most teachers had observed students purchasing food outside school, with 75.4% reporting this “sometimes” or “often” (Table 2). Subject teachers more frequently reported “often” (21.5% vs. 6.8%), while primary teachers more commonly reported “rarely” or “never” (χ^2^(4) = 43.90, *p* < 0.001). Differences by work experience were also significant (χ^2^(8) = 37.24, *p* < 0.001), with more experienced teachers reporting less frequent observation.

Responses to the multi-select item on foods brought from home also took categorical form. Teachers could choose multiple options. Selections are summarized in Table 3. Homemade meals were rarely observed overall (13.6% of yes cases), but affirmative responses were highest in Maikuduk (55.0%) versus only 3.0% in South-East (χ^2^ = 11.044, df = 2, *p* = 0.004). Sandwiches (37.6%) followed a similar gradient, peaking in Maikuduk (48.4%, *p* = 0.021). Fruits (31.0%) showed lower prevalence in South-East (8.8%) than Maikuduk (49.1%, *p* = 0.031). Soda (49.6%) was most reported in New City (54.0%; *p* = 0.002). Fast food (13.7%) and sweets (80.7%, being the most brought item) had no district difference (*p* > 0.05).

### 3.2. Educational Practices and Resources

A substantial proportion of teachers incorporated nutrition and health topics into their teaching: 41.3% (n = 304) conducted class hours regularly, 29.5% (n = 217) did so occasionally, and 29.2% (n = 215) never addressed these topics. Primary school teachers were expected to conduct such activities more regularly (88.6% vs. 12.3% among subject teachers), while nearly half of subject teachers (47.1%) reported never conducting them (Table 4).

Regarding methodological materials on healthy eating, 61.1% (n = 450) reported using them, 15.1% (n = 111) wanted them but did not have access, and 23.8% (n = 175) did not need them. Availability was strongly associated with conducting nutrition and health classes (χ^2^(4) = 684.46, *p* < 0.001). Nearly all teachers who taught nutrition regularly used such materials (99.7%), compared to 66.8% of those who taught occasionally and only 0.9% of those who never taught nutrition. Conversely, 79.5% of non-teaching teachers reported not needing materials, while 19.5% desired access.

Most teachers attributed only a moderate (44.8%) or minor (40.8%) role to schools in shaping student dietary habits, with only 10.5% selecting “very significant.” Perceived parental nutrition knowledge was rated as average by 76.1% of respondents. Primary teachers were more confident in parental knowledge (88.6% rated it “average”) compared to subject teachers, who more frequently selected “hard to say” (22.8% vs. 5.0%).

The top three suggested measures by school teachers to improve school nutrition were improving the school menu (n = 530), educating children (n = 325), and upgrading kitchen equipment (n = 463). Involving parents (n = 189) and training canteen staff (n = 171) were less prioritized.

### 3.3. Knowledge of Nutrition Principles

For all teachers in our study the average total knowledge score of nutrition principles (questions 11A–20A) was 6.26 ± 2.64 out of 12. According to the predefined scale, 23.6% (n = 174) of teachers had low knowledge, 59.9% (n = 441) satisfactory knowledge, and 16.4% (n = 121) good knowledge.

Table 5 summarizes mean scores by key subgroups. Primary school teachers showed higher nutrition knowledge than subject teachers (7.27 ± 2.89 vs. 5.64 ± 2.27). An independent samples *t*-test confirmed this difference. The t-value reached 8.504 (df = 734), and the *p*-value fell below 0.001. Levene’s test indicated unequal variances (F = 45.128, *p* < 0.001), so the adjusted *t*-test results were used.

**Figure 1:**
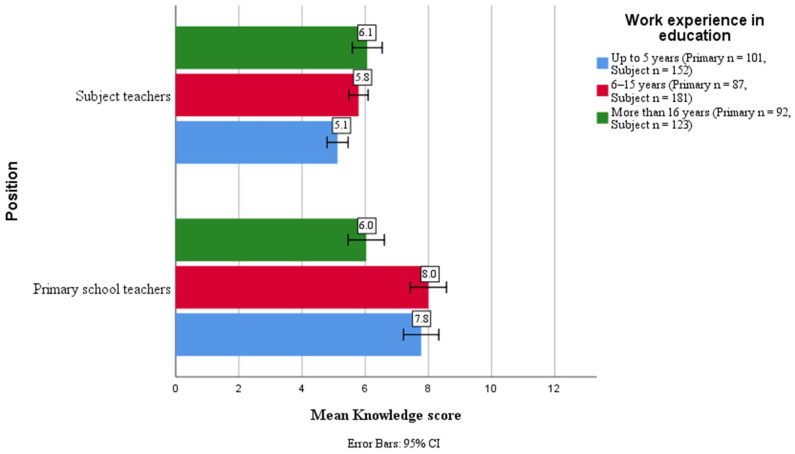
Public school teachers’ mean nutrition knowledge by position and experience.

Figure 1 presents mean knowledge scores by teacher position and work experience. Primary school teachers maintained higher scores across all experience categories.

Performance on individual knowledge items varied considerably (Table 6). Correct responses exceeded 80% for daily fruit and vegetable intake (82.9%), the building function of nutrients (85.1%), and the safety of drinking with meals (82.6%). There were fewer than 40% correctly answered questions on food group priority (40.9%), nutrition-related diseases (29.6%), the fruits myth (22.6%), and eating after 18:00 (38.5%). The most common misconceptions included ranking meat and fish products too highly in the food pyramid (37.2%), believing late dinner inevitably causes weight gain (33.8%), and selecting anorexia as a nutrition-related disease (7.5%).

### 3.4. Predictors of Low Nutritional Knowledge

In the multivariable logistic regression model (Table 7), regular pedagogical engagement in nutrition was associated with substantially lower odds of having low nutrition knowledge compared to a complete lack of such teaching practice (adjusted OR = 0.10, 95% CI 0.05–0.23, *p* < 0.001). Occasional integration of nutrition topics was also associated with a decreased likelihood of low knowledge scores (adjusted OR = 0.41, 95% CI 0.26–0.64, *p* < 0.001). Having 6–15 years of professional experience was associated with lower odds of poor knowledge compared to having over 16 years of experience (adjusted OR = 0.47, 95% CI 0.29–0.77, *p* = 0.003). Geographic district variations remained statistically significant after adjustment, compared to the South-East district, educators in the New City district showed higher odds of low knowledge (adjusted OR = 2.49, 95% CI 1.36–4.56, *p* = 0.003), whereas those in Maikuduk had lower odds (adjusted OR = 0.66, 95% CI 0.44–1.00, *p* = 0.048). Teaching position did not remain a statistically significant predictor in this adjusted dichotomized model. The final model had acceptable goodness-of-fit (Hosmer–Lemeshow *p* = 0.527, Nagelkerke R^2^ = 0.227) and correctly classified 76.1% of the cases.

Furthermore, applying an alternative, stricter cut-off threshold for the logistic categorization of ‘low knowledge’ (defined as a score of ≤5 rather than ≤4) did not alter the direction or statistical significance of the primary model. Under this alternative threshold, regular pedagogical engagement in nutrition remained a robust protective factor against poor knowledge scores (adjusted OR 0.30, 95% CI 0.18–0.48, *p* < 0.001), further reinforcing the reliability of the dichotomized approach.

To verify the robustness of the dichotomized model, a prespecified sensitivity analysis was conducted using a multiple linear regression framework with the continuous 12-point nutrition knowledge score as the dependent variable. The findings remained highly consistent with the primary analysis. The lack of pedagogical practice in nutrition remained a robust, statistically significant predictor of lower overall knowledge scores (B = −1.40, 95% CI −1.86 to −0.94, *p* < 0.001). Teaching at the secondary level, compared to primary education, was independently associated with a nearly one-point reduction in the continuous knowledge score (B = −0.96, *p* < 0.001). Neither years of work experience nor school location significantly predicted continuous knowledge scores, reinforcing the reliability of the initial logistic regression approach.

Although mean knowledge scores did not differ significantly across districts in crude ANOVA comparisons, district-level associations emerged in the adjusted logistic regression model.

## 4. Discussion

This study extends the literature on school-based nutrition education by examining teachers not only as knowledge holders but as functional actors within the school food environment. The findings indicate that teacher knowledge in this urban Kazakh context is moderate overall, yet uneven in structure. While many respondents had adequate understanding of basic dietary recommendations, applied knowledge related to common nutrition myths was substantially weaker. This pattern suggests that cognitive familiarity with guidelines does not necessarily translate into critical evaluation of circulating dietary claims, a distinction that has practical implications in contemporary information environments.

### 4.1. School Teachers’ Nutrition Knowledge

The mean nutrition knowledge score of 6.26 ± 2.64 out of 12 (52.2%) places the sample within the “satisfactory” range, with only 16.4% reaching the “good” category and nearly a quarter (23.6%) classified as having low knowledge. Comparable deficits have been documented among school teachers in Bangladesh (54.5% correct responses), Kuwait (61.9%), Brazil (41%), and Turkey (80.9% classified as inadequate) [23,24,31,32], suggesting that moderate nutrition knowledge among school educators is a consistent pattern across diverse national contexts rather than a finding specific to Kazakhstan.

Item-level analysis revealed uneven performance across question types. Teachers showed stronger results on basic factual items, such as recommended fruit and vegetable intake and nutrient functions, while performing substantially worse on questions addressing common dietary myths. This pattern indicates gaps in applied nutrition knowledge rather than deficits in general critical thinking, as the instrument was designed to assess content knowledge rather than reasoning skills. These results corroborate prior research showing that even trained educators may perpetuate common food myths due to exposure to unreliable or oversimplified sources [18,33], and are consistent with broader evidence that repeated exposure to misleading nutritional information in the media environment can reinforce erroneous beliefs, particularly when individuals lack consistent access to evidence-based sources [19,33,34,35].

Among the specific misconceptions identified, incorrect responses regarding eating after 6:00 p.m. (33.8%; Item 20A) and gluten avoidance (31.0%; Item 19A) were similarly prevalent. The persistence of the late-eating myth likely reflects its intuitive appeal as a weight management heuristic, while misconceptions about gluten are plausibly amplified by social media promotion of gluten-free diets as universally beneficial regardless of medical indication [33]. Comparable misconceptions, including confusion about carbohydrates, fats, and dietary patterns associated with chronic disease, have been found among teachers and education students in other countries [36,37,38].

Perhaps the most notable pattern concerned the relationship between knowledge and pedagogical practice. Primary school teachers, who are structurally responsible for integrating general health topics into the curriculum, scored significantly higher than subject teachers (mean 7.27 vs. 5.64, Cohen’s d = 0.53, *p* < 0.001). This effect size reflects practical as well as statistical relevance. Primary teachers also reported the most active engagement in nutrition education, with 88.6% regularly conducting health-related class sessions. Taken together, these data indicate that regular engagement with nutrition content is associated with higher knowledge scores, and that the nature of pedagogical practice rather than job title alone may account for observed group differences [30,34]. However, the cross-sectional design does not permit conclusions about the direction of this association. Teachers with greater baseline knowledge or personal interest in nutrition may be more likely to deliver nutrition-related content, while regular engagement may also reinforce existing knowledge.

### 4.2. Factors Associated with Low Levels of Nutrition Knowledge

Our multivariable models identified regular pedagogical practice as a strong protective factor. Specifically, the regular teaching of nutrition topics was associated with a substantially lower likelihood of possessing poor nutrition literacy (OR 0.10). It is consistent with international evidence that nutrition and food literacy are more commonly embedded in primary curricula and that primary teachers often see themselves as having a broader pastoral and health-promoting role [13,39,40]. The strong association between regular delivery of nutrition-related lessons and lower odds of inadequate knowledge highlights the interplay between professional practice and cognitive preparedness. Due to the cross-sectional design, the direction of this relationship cannot be determined. Teachers with higher baseline knowledge may be more inclined to conduct nutrition education, while repeated engagement in such activities may reinforce knowledge retention. Rather than implying causality, the findings indicate that pedagogical engagement and knowledge appear closely linked within the institutional setting.

Teachers with 6–15 years of experience were less likely to have low nutrition knowledge than those with more than 16 years of experience. One explanation is that mid-career teachers may remain more engaged in continuing education and school-based initiatives, while more experienced educators may rely on established routines and consult newer resources less often. This interpretation requires caution, since teachers’ learning needs vary and pedagogical approaches may not differ meaningfully across experience levels [41,42]. Still, the finding aligns with evidence suggesting that sustained engagement in nutrition-related teaching and repeated practice contribute to stronger competence over time, particularly when educators remain actively involved in curriculum delivery and professional development [43,44].

The persistence of the district variable in the multivariable model indicates a statistical association between school location and knowledge levels. The mechanisms underlying this association were not directly measured in this study. Hypothetically, inter-district differences could reflect variations in the availability of teaching materials, institutional capacity, or characteristics of the working environment, but such explanations remain speculative without supporting data. International research documented territorial disparities, showing that schools in different city areas often have unequal access to resources and organizational support [45]. A systematic review noted that the food environment around schools often features high availability of unhealthy food and that these patterns correlate with the socioeconomic profile of districts, which indirectly limits schools’ ability to implement healthy eating programs [46]. Another study showed that nutrition policies alone do not yield equal effects across schools, as outcomes depend heavily on resource availability and structural support, with territorial and social variables acting as modifiers [47].

### 4.3. Teachers’ Perceptions of School Nutrition

Primary school teachers rated school meal quality significantly higher than subject teachers (mean 4.26 vs. 3.96, *p* < 0.001). This difference may reflect varying levels of professional involvement during meal times, as primary teachers more frequently accompany students to the cafeteria. However, since our study did not include objective assessments of menus or meal composition, this interpretation remains plausible rather than demonstrated. International studies confirm that educators’ involvement in the school food environment substantially influences both their subjective evaluations and students’ eating behaviors [48,49].

The overall positive assessment of school meals may reflect the initial implementation of recent nutritional reforms in Kazakhstan. Updates to national sanitary and hygienic standards, guided by World Health Organization recommendations, have mandated an increased proportion of vegetables, fruits, and dairy products in school diets, alongside reductions in sugar, salt, and saturated fats [10]. However, interpreting these high subjective ratings requires caution due to potential social desirability bias. Although the survey was anonymous, teachers evaluated the food environments of their employing institutions, which may have suppressed critical assessments. This dynamic partially explains the discrepancy between the high mean quality scores and concurrent reports from the same educators regarding students frequently rejecting cafeteria food in favor of less healthy, off-campus alternatives. Also, this divergence likely reflects differing evaluation paradigms: adults generally assess school meals based on institutional compliance and nutritional adequacy, whereas adolescents prioritize taste preferences. In this case, the reported ratings function as indicators of teacher perception rather than objective metrics of menu quality. Nevertheless, these observations align with recent national data highlighting persistent challenges in menu acceptability. A UNICEF-supported Childhood Obesity Surveillance Initiative study reported that only 59% of Kazakh children approved of the school menu, while 21% disliked it and 20% consumed no school food at all [50]. These figures are broadly consistent with the meal refusal patterns observed by the educators in our sample.

These findings align with FAO data indicating that a substantial proportion of Kazakh schoolchildren purchase sugary drinks and snacks daily [51,52], and are consistent with international evidence on competitive food environments around schools [53,54].

Primary school teachers, who interact more intensively with both children and parents, appeared somewhat more confident in perceived parental knowledge than subject teachers, who more frequently selected “hard to say.” This cautious view of the school’s influence is noteworthy given that international policy frameworks, including the Health-Promoting Schools model and WHO recommendations, explicitly position schools as central platforms for improving child nutrition and preventing non-communicable diseases [55].

Strengthening cafeteria oversight and teacher involvement could enhance trust in school meals and support broader nutrition reform efforts. International experience suggests that such measures yield tangible benefits: in Italy, national school nutrition standards combined with increased parental involvement in quality control led to higher satisfaction and greater trust in service providers [56]. Given teachers’ moderate quality ratings, frequent observations of student refusals, and the competitive pressure from unhealthy alternatives, similar comprehensive approaches may be acceptable in the Kazakh context.

### 4.4. Implications for Kazakhstan’s School Feeding and Nutrition Policies

Kazakhstan has made measurable progress in institutionalizing school nutrition standards, with recent regulatory updates mandating higher proportions of vegetables, fruits, and dairy while restricting sugar, salt, and ultra-processed foods [57]. FAO-supported pilot programs are advancing local procurement and menu diversification [58]. Within this policy landscape, the present findings identify teachers as an underused resource. Nearly one-third of teachers reported no integration of nutrition or health topics into classroom instruction, a substantial subset showed low applied knowledge, and many expressed cautious views of schools’ influence on dietary behavior.

These patterns point to a gap between structural reforms in menu composition and the professional capacity of educators to reinforce those reforms in daily school life. Subject teachers, who constitute approximately 62% of the sample, were markedly less engaged in school-based nutrition initiatives and scored lower on the knowledge assessment. Addressing this requires purpose-built instructional materials designed for cross-curricular integration in secondary disciplines, such as biology and chemistry, alongside continuing professional development that specifically addresses dietary misconceptions. These measures need not impose substantial administrative demands if modules are concise, pre-designed, and accessible through existing digital platforms.

The findings are not specific to Kazakhstan. Many countries have strengthened school meal standards while investing less in the educational workforce that mediates those standards in practice. Where teachers lack consistent nutrition knowledge, the impact of policy reforms on students’ dietary understanding and behavior may be attenuated. In settings undergoing dietary transition, teachers represent a scalable channel for reinforcing evidence-based norms that extend beyond the cafeteria.

### 4.5. Strengths

This study has several notable strengths. The sample size of 736 teachers across 12 schools in three socioeconomically diverse urban districts provides reasonable statistical power and within-city contextual diversity across distinct urban settings. The response rate of 92% reduces concerns about non-response bias. The use of a structured KAP-based questionnaire with content expert review, pilot testing, and acceptable internal consistency (α = 0.70) supports the interpretability of findings. The inclusion of both primary and subject teachers allowed meaningful within-sample comparisons, and the multivariable regression approach enabled adjustment for key confounders.

Future research should adopt longitudinal designs to clarify temporal relationships. Mixed-method approaches may further clarify how institutional context and professional norms shape teachers’ engagement with school nutrition initiatives.

### 4.6. Limitations

Several methodological limitations must be acknowledged. First, the cross-sectional design precludes the establishment of causal inference. Specifically, the strong inverse association between active pedagogical practice in nutrition and poor knowledge scores may reflect reverse causality, because educators with a pre-existing personal interest or superior baseline knowledge in health topics may selectively choose to integrate these concepts into their teaching routines. Second, the reliance on self-reported data within an institutional setting introduces vulnerability to social desirability bias. Teachers may have experienced implicit pressure to rate employer-provided school meals favorably. This institutional loyalty likely explains the inconsistency between these high subjective ratings and the educators’ concurrent reports of frequent student meal refusal. These perceptions were not supported by objective assessments of meal quality or spatial mapping of nearby food outlets, which restricts a comprehensive evaluation of the surrounding food environment. Third, our models did not account for several potentially relevant variables, including demographic factors (age, sex), prior formal nutrition training, educators’ primary information sources, and family-level influences on student habits, all of which could act as unmeasured confounders influencing knowledge scores and adherence to dietary myths. From a statistical and psychometric perspective, the dichotomization of knowledge scores, although clinically motivated, reduced statistical power and may have attenuated the observed associations. Additionally, while pilot testing supported item clarity and internal consistency, the knowledge categories serve exploratory and descriptive purposes rather than acting as externally validated diagnostic thresholds. The questionnaire covered multiple conceptual domains within the KAP framework, and its attitudinal and practice items were not designed to form unidimensional scales.

Another limitation concerns the handling of the nested data structure. Although the sampling design explicitly accounted for clustering, through pre-specified design effect estimation (DEFF = 2.18, ρ = 0.02) that inflated the required sample size accordingly, the inferential analyses (*t*-tests, ANOVA, logistic regression) did not employ multilevel modelling or cluster-robust standard errors. This approach may underestimate standard errors, potentially inflating the precision of reported associations. Future studies in this setting should use multilevel regression frameworks to formally partition between-school and within-school variance. Additionally, unequal group sizes across districts reduce the statistical power of post hoc comparisons and warrant caution when interpreting district-level variations.

## 5. Conclusions

This cross-sectional survey of 736 public school teachers in Karaganda, Kazakhstan, found that nutrition knowledge was satisfactory on average, with nearly one quarter of teachers scoring in the low category. Persistent gaps were identified in applied understanding of common dietary myths. Higher knowledge scores were observed among primary school teachers and those who regularly delivered nutrition-related lessons, though the cross-sectional design precludes causal conclusions; teachers with stronger baseline knowledge may self-select into nutrition education activities, or regular engagement may reinforce existing knowledge. These findings contribute to the limited evidence base on teacher nutrition competencies in Central Asia. It is highly recommended to prioritize the development of specialized, cross-curricular instructional materials and continuing professional development modules adapted specifically for secondary educators. Equipping all teachers with evidence-based nutrition knowledge is a foundational step toward establishing sustainable healthy eating behaviors among students at national and regional levels.

## Figures and Tables

**Table 1:** Demographic characteristics of participants (n = 736).

Variable	Category	n (%)
Position	Primary school teacher	280 (38.0)
	Subject teacher	456 (62.0)
Work experience	≤5 years	253 (34.4)
	6–15 years	268 (36.4)
	>16 years	215 (29.2)
School district	South-East	72 (9.8)
	New City	355 (48.2)
	Maikuduk	309 (42.0)

**Table 2:** Teachers’ perceptions of the school food environment (n = 736).

Variable	Category	n (%)
Quality of food products and meals (1 = very low, 5 = very high)	1 point	1 (0.1)
	2 points	4 (0.5)
	3 points	76 (10.3)
	4 points	511 (69.4)
	5 points	144 (19.6)
Frequency of students refusing school meals	Almost never	103 (14.0)
	Sometimes	292 (39.7)
	Often	61 (8.3)
	Almost always	8 (1.1)
	Don’t know/Don’t observe	272 (37.0)
Observation of children buying food outside school	Yes, observe it often	117 (15.9)
	Notice it sometimes	438 (59.5)
	Rarely	102 (13.9)
	Never noticed	57 (7.7)
	Hard to say	22 (3.0)

**Table 3:** Foods teachers observed students bringing to school (multi-select, n = 736).

Food Type	n Selecting (%)
Homemade food (e.g., main dish, salad, sliced vegetables, syrniki)	100 (13.6)
Fast food	101 (13.7)
Fruits	228 (31.0)
Sandwiches	277 (37.6)
Chips/crackers	336 (45.7))
Soda/sweet drinks	365 (49.6)
Sweets, chocolate, candies	594 (80.7)
Other	1 (0.1)

**Table 4:** Frequency of conducting nutrition and health classes (n = 736).

Frequency	Overall n (%)	Primary Teachers n (%)	Subject Teachers n (%)
Yes, regularly	304 (41.3)	248 (88.6)	56 (12.3)
Sometimes	217 (29.5)	32 (11.4)	185 (40.6)
No, do not	215 (29.2)	0 (0.0)	215 (47.1)

**Table 5:** Mean nutrition knowledge scores by teacher position, work experience, and district.

Group	Subgroup	Mean Score (SD)	n
Position	Primary	7.27 (2.89)	280
	Subject	5.64 (2.27)	456
	≤5 years	6.18 (2.73)	253
Work experience	6–15 years	6.51 (2.50)	268
	>16 years	6.05 (2.70)	215
	South-East	5.36 (2.85)	72
District	New City	6.36 (2.50)	355
	Maikuduk	6.36 (2.72)	309

Note: One-way ANOVA: work experience F(2, 733) = 1.962, *p* = 0.141 (non-significant); district F(2, 733) = 4.687, *p* = 0.009. Games–Howell post hoc: South-East vs. New City mean difference −1.00, *p* = 0.019; South-East vs. Maikuduk mean difference −1.00, *p* = 0.021; New City vs. Maikuduk *p* = 1.000. Independent samples *t*-test (position): t(734) = 8.504, *p* < 0.001, Levene’s F = 45.128, *p* < 0.001.

**Table 6:** Performance on individual knowledge items (n = 736).

Question	Correct Answers, n (%)	Most Common Incorrect Answer (%)	Don’t Know (%)
11A (Daily fruit/veg intake)	610 (82.9%)	100–399 g (16.3%)	0.4
12A (Food group priority)	301 (40.9%)	Meat and fish products ranked high (37.2%)	0.0
13A (Main energy source)	559 (76.0%)	Vitamins (11.5%)	2.3
14A (Nutrients build function)	626 (85.1%)	Vitamins (4.8%)	3.8
15A (Alimentary diseases)	526 (71.5%)	Hereditary chronic diseases (16.4%)	11.0
16A (Nutrition-related diseases)	218 (29.6%)	Anorexia selected (7.5%)	1.0
17A (Drinking with meals)	608 (82.6%)	Drinking helps move the food bolus through the esophagus (6.9%)	6.4
18A (Fruits myth)	166 (22.6%)	Do not know (5.3%)	5.3
19A (Gluten myth)	508 (69.0%)	Excluding gluten without medical reason may cause micronutrient deficiency (12.4%)	10.5
20A (Eating after 18:00)	283 (38.5%)	Late dinner inevitably causes weight gain due to slowed metabolism (33.8%)	3.5

**Table 7:** Multivariable logistic regression predicting low nutrition knowledge among teachers (adjusted odds ratios).

Predictor	Category (vs. Reference)	OR	95% CI	*p*
Nutrition education	Regularly vs. No	0.104	[0.048, 0.225]	<0.001
	Sometimes vs. No	0.410	[0.262, 0.640]	<0.001
Position	Subject vs. Primary	1.201	[0.600, 2.402]	0.605
Work experience	≤5 years vs. >16 years	1.089	[0.684, 1.731]	0.720
	6–15 years vs. >16 years	0.470	[0.287, 0.770]	0.003
District	New City vs. South-East	2.489	[1.358, 4.562]	0.003
	Maikuduk vs. South-East	0.662	[0.440, 0.997]	0.048

## Data Availability

Data supporting the reported results are not publicly available due to privacy and ethical restrictions protecting participant confidentiality. Researchers may contact the corresponding author for access to anonymized data, subject to ethical approval.

## Abbreviations

The following abbreviations are used in this manuscript:

DEFF	The design effect
ME	Margin of error
KAP	Knowledge–Attitudes–Practice
ANOVA	Analysis of variance
CI	Confidence interval
M	Mean
OR	Odds ratio
SD	Standard deviation
UNICEF	United Nations International Children’s Emergency Fund
WHO	World Health Organization
FAO	Food and Agriculture Organization

## Appendix A

Teacher Nutrition Knowledge, Attitude, and Practice (TN-KAP) Questionnaire

Part 1_Attitudes and Practices

1A. Please indicate your position:

☐ Primary school teacher
☐ Subject teacher

2A. How do you rate the quality of food products and prepared meals provided in the school cafeteria? (1—very low, 5—very high)

☐ 1 point
☐ 2 points
☐ 3 points
☐ 4 points
☐ 5 points

3A. Based on your observations, how often do students refuse school meals?

☐ Almost never
☐ Sometimes
☐ Often
☐ Almost always
☐ I don’t know/I don’t observe

4A. Have you observed children buying food products outside of school during breaks, before, or after lessons (e.g., fast food, chips, sweets)?

☐ Yes, I observe it often
☐ I notice it sometimes
☐ Rarely
☐ I have never noticed
☐ Hard to say

5A. Based on your observations, which food products do students most often bring with them to school?

☐ Homemade food (e.g., a main dish, salad, sliced vegetables, syrniki)
☐ Sandwiches
☐ Fast food
☐ Chips/Crackers
☐ Sweets, chocolate, candies
☐ Fruits
☐ Soda/Sweet drinks
☐ Other: __________

6A. Do you conduct class hours or activities dedicated to the topic of nutrition and health?

☐ Yes, regularly
☐ Sometimes
☐ No, I do not

7A. Do you have methodological materials for developing healthy eating habits in schoolchildren?

☐ Yes, I use them
☐ No, but I would like to have them
☐ No, I do not need them

8A. In your opinion, what role does the school play in shaping children’s eating behaviors and habits?

☐ Very significant
☐ Moderate
☐ Insignificant
☐ No influence at all
☐ Hard to say

9A. In your opinion, what is the level of awareness of your students’ parents regarding the principles of healthy eating?

☐ High
☐ Average
☐ Low
☐ Hard to say

10A. In your opinion, what measures could help improve student nutrition?

☐ Improving the menu
☐ Upgrading the kitchen equipment
☐ Involving parents
☐ Improving the competence of cafeteria staff
☐ Educating children
☐ Other: __________

Part 2_Knowledge (correct answers highlighted)

11A. How many grams of fresh vegetables and fruits do you think should be consumed daily? (1 point)

☐ Less than 250 g
☐ **250–399 g**
☐ At least 400 g
☐ Hard to say

12A. Which food groups should predominate in the daily diet? Rank the products on a 5-point scale, from 1 to 5, where 1 is the smallest quantity and 5 is the largest quantity. Use each number only once. (1 point) *(The correct ranking would be: **5-Vegetables/fruits, 4-Whole-grains, 3-Protein, 2-Dairy, 1-Oils**)*

☐ Vegetables, fruits, greens
☐ Whole-grain products
☐ Protein products (fish, poultry, legumes)
☐ Dairy products
☐ Vegetable oils

13A. What do you think is the main source of energy for the body? (1 point)

☐ Proteins
☐ Fats
☐ **Carbohydrates**
☐ Vitamins
☐ Minerals
☐ Hard to say

14A. Which nutrient primarily performs a building (structural) function in the body? (1 point)

☐ **Proteins**
☐ Fats
☐ Carbohydrates
☐ Vitamins
☐ Minerals
☐ Hard to say

15A. What is understood by diet-related diseases? Choose one answer. (1 point)

☐ **Diseases arising from nutritional disorders, including deficiency or excess of nutrients**
☐ Infectious diseases transmitted by food and caused by bacteria
☐ Hereditary chronic diseases caused by genetic disorders
☐ Chronic diseases arising from genetic mutations
☐ Hard to say

16A. Which diseases can be a consequence of an unbalanced diet? Choose one or more correct answers. (2 points)

☐ **Obesity**
☐ **Type 2 diabetes**
☐ Anorexia
☐ **Atherosclerosis**
☐ **Gallstone disease**
☐ Stomach ulcer
☐ Hard to say

17A. Which statement about drinking liquid with meals is incorrect? Choose one answer. (1 point)

☐ **Liquid “dilutes” gastric juice and interferes with normal digestion**
☐ In a healthy person, liquids quickly pass into the small intestine and are not retained in the stomach
☐ Moderate consumption of water during meals does not harm health and digestion
☐ Drinking liquid with food can be useful for better movement of the food bolus
☐ Hard to say

18A. Which statement about fruits is incorrect? Choose one or more correct answers. (2 points)

☐ Fruits contain vitamins and dietary fibers necessary for health
☐ The daily diet should include a variety of vegetables and fruits
☐ **Fruits are harmful to health due to their high sugar content**
☐ **Unlimited consumption of fruits is part of a balanced diet**
☐ Hard to say

19A. Which statement about gluten is incorrect? Choose one answer. (1 point)

☐ Eliminating gluten without medical indications can lead to micronutrient deficiencies
☐ Whole-grain products containing gluten can support the microbiota due to dietary fibers
☐ **Gluten “clogs” the intestines and blood vessels, provokes inflammation, and causes weight gain**
☐ Gluten-free products may contain less fiber and more sugar
☐ Hard to say

20A. Which statement about eating after 6:00 PM corresponds to scientific data? Choose one answer. (1 point)

☐ A late dinner inevitably leads to weight gain due to a slowed metabolism
☐ **Eating after 6:00 PM is permissible if the daily calorie balance is maintained**
☐ The digestive system stops its active work after 6:00 PM
☐ Any food intake after 6:00 PM leads to fat deposition regardless of the diet
☐ Hard to say

**Part 3_Demographics**

21A. In which district is the educational institution where you work located?

☐ Yugo-Vostok (South-East)
☐ Novy Gorod (New City)
☐ Maikuduk

22A. Your work experience in an educational institution:

☐ Up to 5 years
☐ 6–15 years
☐ More than 16 years

**Knowledge subscale. Scoring system and answer key**

The knowledge subscale comprised ten items (Q11A–Q20A) with a maximum total score of 12 points. Three distinct scoring rules were applied depending on item format.

**Single-answer items (1 point each; 7 items: Q11A, Q13A, Q14A, Q15A, Q17A, Q19A, Q20A).** One point was awarded for selecting the single correct response. No partial credit was available.

**Food group ranking item (1 point; Q12A).** One point was awarded only when the respondent’s ranking of five food groups exactly matched the reference order in its entirety. The reference ranking, derived from current national and WHO dietary guidelines, was as follows (from largest to smallest recommended proportion): (1) vegetables, fruits, and greens—5 points; (2) whole-grain products—4 points; (3) protein products (fish, poultry, legumes)—3 points; (4) dairy products—2 points; (5) vegetable oils—1 point. Any deviation from this exact sequence was scored as 0.

**Multiple-answer items (2 points each; 2 items: Q16A, Q18A).** Two points were awarded only when all correct options were selected simultaneously, with no incorrect options included and no omissions. Partial credit was not available. This all-or-nothing rule was applied to increase the discriminative capacity of the scale.

—*Q16A (diseases caused by an unbalanced diet):* the correct response required selecting all four of the following: obesity, type 2 diabetes, atherosclerosis, and gallstone disease. Selecting anorexia or stomach ulcer, or omitting any correct option, resulted in a score of 0.

—*Q18A (incorrect statements about fruits):* the correct response required selecting both of the following incorrect statements: “fruits are harmful to health due to their high sugar content” and “unlimited consumption of fruits is part of a balanced diet.” Selecting only one, or including any other option, resulted in a score of 0.

**Score categorization.** For descriptive purposes, total scores were classified as: low (0–4 points), satisfactory (5–8 points), and good (9–12 points). These thresholds were defined a priori by dividing the score range into approximate tertiles, consistent with approaches used in comparable KAP-based studies, and were confirmed as interpretively reasonable during pilot testing. They serve as descriptive categories for group-level analysis and do not constitute externally validated clinical thresholds. For multivariable analysis, knowledge was dichotomized as low (0–4) versus satisfactory or good (≥5).
